# The development of low‐calorie sugar and functional jujube food using biological transformation and fermentation coupling technology

**DOI:** 10.1002/fsn3.963

**Published:** 2019-03-06

**Authors:** Yan Men, Ping Zhu, Yueming Zhu, Yan Zeng, Jiangang Yang, Yuanxia Sun

**Affiliations:** ^1^ National Engineering Laboratory for Industrial Enzymes Tianjin Institute of Industrial Biotechnology Chinese Academy of Sciences Tianjin China

**Keywords:** D‐allulose, enzyme transformation, jujube juice, lactic acid bacteria, probiotic fermentation

## Abstract

Jujube juice has been used as ingredient in a range of foods and dietary supplements. In this study, an enzyme transformation and fermentation coupling technology was applied to increase the nutritional value of concentrated/extracted Jinsi jujube juice. Two enzymes, D‐glucose isomerase (GI) and D‐allulose 3‐epimerase (DAE), were employed to convert the glucose and fructose to a low‐calorie sweeter D‐allulose with a concentration of 110 g/L in jujube juice. Furthermore, the mixed cultures of *Pediococcus pentosaceus* PC‐5 and *Lactobacillus plantarum* M were employed to increase the content of nutrition components related to bioactivities and flavor volatiles in jujube juice. Accordingly, this fermentation accumulated 100 mg/L gamma‐aminobutyric acid (GABA), which has neurotransmission, hypotension, diuretic, and tranquilizer effects, and increased the content of branched‐chain amino acids (BCAAs) and many free amino acids (Asp, Glu, Gly, and Ala) at different level. The fermentation not only maintained the concentration of native functional components such as cyclic adenosine monophosphate (cAMP) and minerals, but also increased the content of iron (Fe^2+^) and zinc (Zn^2+^), which have blood and eyesight tonic function. The value‐added jujube juice might serve as a low‐calorie and probiotic functional beverage and show high application potential in food industry.

## 1 INTRODUCTION

Jujube (*Ziziphus jujuba* Mill), a thorny rhamnaceous plant, is commonly known as Chinese jujube or red date. Jujube fruit contain lots of nutritional constituents such as polysaccharide, triterpenoid, flavonoids, vitamins, cyclic nucleotide, and phenolic compounds (Du et al., 2013; Lee, Min, Lee, Kim, & Kho, 2003; Li, Fan, Ding, & Ding, 2007). Specially, jujube fruits contain a certain amount of cyclic adenosine monophosphate (cAMP) which has positive effect on the heart muscle, nutritional myocardium, diastolic blood vessels, antiarrhythmia, and antiplatelet aggregation (Beavo & Brunton, 2002). Developing functional foods being rich in cAMP has great economic potential and market value. Jujube has also been used as a traditional Chinese medicine (TCM) for thousands of years with its numerous health‐promoting effects such as anti‐inflammatory (Yu et al., 2012), anticancer (Plastina et al., 2012), gastrointestinal protective (Huang, Yen, Sheu, & Chau, 2008), antioxidant (Cheng, Zhu, Cao, & Jiang, 2012), anti‐insomnia, and neuroprotective effects (Yoo et al., 2010).

Although the jujube fruit has high nutritional value and numerous bioactivities, it contains high content of high‐calorie sugar, such as sucrose, D‐glucose, and D‐fructose. D‐Allulose as a low‐calorie sweetener has been approved by the US Food and Drug Administration (FDA) to be “generally recognized as safe” (GRAS) and is allowed to be used as an ingredient in a range of foods and dietary supplements (FDA 2011). Oral administration of D‐allulose will induce GLP‐1 release and activate vagal afferent signaling, resulting in reduce in food intake and promotion glucose tolerance in healthy and obese diabetic (Iwasaki et al., 2018). It has various specific nutritional and biological functions, such as protecting pancreas beta‐islets (Hossain et al., 2012), improving insulin sensitivity and glucose tolerance (Hossain et al., 2011), reducing intra‐abdominal fat accumulation (Matsuo & Izumori, 2009), scavenging reactive oxygen species activity (Murata et al., 2003), neuroprotective effects on 6‐hydroxydopamine‐induced apoptosis (Takata et al., 2005), and lowering abdominal fat accumulation (Matsuo et al., 2001). Enzymatic transformation of D‐fructose into D‐allulose has been achieved by using D‐allulose 3‐epimerase. This reaction would decrease the content of D‐glucose and D‐fructose in jujube juice and obtain a “healthy sugar” juice.

Lactic acid bacteria fermentation technology has been proved to be an effective way to maintain or improve the flavor, safety, nutrition, quality, and shelf life of fruits and vegetables. The nutritional value of fermented foods increased through accumulation of free amino acids (FAAs) during fermentation process. Typically, some lactic acid bacteria produce γ‐aminobutyric acid (GABA) which has neurotransmission, hypotension, diuretic, and tranquilizer effects (Wong, Bottiglieri, & Snead, 2003). The development of GABA‐enriched beverages using fermentation method has succeeded in black raspberry juice (Kim, Lee, Ji, Lee, & Hwang, 2009), milk (Nejati et al., 2013), and tempeh‐like beverage (Aoki, Furuya, Endo, & Fujimoto, 2003). In addition, lactic acid bacteria as probiotics are increasingly used as food supplements. The probiotic products are usually marketed in the forms of fermented dairy products, fruit, or vegetable juices (carrot juice, tomato juice) which serve as media for cell growth.

In this study, we attempt to increase the nutritional value of concentrated/extracted jujube juice by coupling enzyme transformation and lactic acid bacteria fermentation. The glucose and fructose content in different kinds of jujube was compared. Two enzymes, GI and DAE, were combined to convert glucose and fructose to a low‐calorie sweeter D‐allulose. Furthermore, the fermentation of jujube juice using two kinds of lactic acid bacteria was carried out to increase the content of GABA and many free amino acids.

## 2 MATERIALS AND METHODS

### 2.1 Enzymes, strains, and materials

D‐allulose 3‐epimerase (DAE) was prepared according to the extra‐cellular production via secretive expression system (Chen et al., 2016). D‐glucose isomerase (GI) was purchased from Novozymes^®^, Ltd in China. The activity of DAE reached 31.0 U/ml, and GI was 400 IGIU/g. Lactic acid bacteria, such as *Pediococcus pentosaceus* PC‐5, *Lactobacillus plantarum* M, *Lactobacillus rhamnosus*, and *Lactobacillus acidophilus*, were stored in our laboratory. Five kinds of jujube fruit used in this paper were collected from September to October of 2016 in Taiyuan city of Shanxi Province, China.

### 2.2 Analysis of sugar content in five jujube fruit

*Ziziphus jujuba* commonly called jujube or Chinese date. In this study, the jujube fruits from five cultivars (Changhong, J‐CH; Dongzao, J‐DZ; Jinsi, J‐JS; Pozao, J‐PZ; and Yuanling, J‐YL) were collected in the autumn. All jujube samples were sliced, boiled in water for 3 hr, and then, the residue was removed to obtain concentrated juice. The content of glucose, fructose, and sucrose was analyzed by high‐performance liquid chromatography (HPLC) equipped with a refractive index detector and one column of Sugar‐Pak™ (6.5 mm × 300 mm; Waters). Total polysaccharide was determined with phenol–sulfuric acid colorimetric method (Cheung et al., 2009). The reducing sugar was detected by DNS method (Cheung et al., 2009).

### 2.3 The enzymatic conversion of monosaccharide in jujube juice

The enzymatic transformation catalyzed by GI and DAE was performed at 55°C in concentrated jujube juice, the pH of which was adjusted to 6.0 using 1 M NaHCO_3_. After reaction for 4 hr, samples were analyzed by HPLC to calculate the concentration of D‐allulose. The effect of temperature (from 50 to 65°C), pH (from 4.0 to 8.0), and enzyme amount (from 0.5 to 2.0 g/L) on D‐allulose conversion was investigated.

### 2.4 Fermentation of jujube juice using lactic acid bacteria

*Pediococcus pentosaceus* PC‐5 and *L. plantarum* M were chosen to evaluate their cell growth, acid production, and content of total polyphenolic and total flavonoids. The selected starters were pre‐cultured in MRS media at 37°C for 24 hr until to approximately 10^8^~10^9^ c.f.u/ml. Then, cells were inoculated into concentrated jujube juice with six times dilution in 500 ml flasks. Fermentation process was then carried out at 37°C for 24 hr. Samples were collected for HPLC and cell growth analysis. Viable cells (c.f.u/ml) were determined by the standard plate count method.

### 2.5 Nutritional ingredient analysis

The concentrations of organic acids (lactic and acetic acid) were quantified by HPLC using an Aminex HPX‐87H column (ion exclusion, BioRad) with an UV detector operating at 210 nm. A mobile phase of 10 mM H_2_SO_4_ solution was used at a flow rate of 0.6 ml/min, and the column was operated at 60°C.

The FAAs and γ‐aminobutyric acid (GABA) were determined by HPLC using a ZORBA Eclipse‐AAA (4.6 mm × 150 mm; Agilent) with an UV detector operating at 338 nm. The mobile phases were A (Equilibrium phase): sodium dihydrogen phosphate (40 mM) and B (Elution phase): acetonitrile:methanol:water (45:45:10). Borate buffer was used as a derivative reagent. The flow rate was 2 ml/min at 40°C.

The concentration of cAMP was also measured by HPLC using a reverse‐phase Ultimate C18 column (21.2 mm × 250 mm, 5 μm particles, Welch, Shanghai, China) with an UV detector operating at 254 nm. A mobile phase of potassium dihydrogen phosphate (20 mM):methanol = 80:20 solution was used at 0.8 ml/min, and the column was operated at 40°C.

Metal ions were determined using atomic absorption spectrophotometer (Spectra‐AA220, Varian Co., Palo Alto, CA, USA) after digestion in mixed acids (nitric acid:perchloric acid = 4:1). Phosphorus content was determined by the molybdenum blue method in the wavelength of 660 nm (Wei, Chen, & Xu, 2009).

The total phenolic content in jujube juice was determined according to the Folin–Ciocalteu colorimetric method (Tawaha, Alali, Gharaibeh, Mohammad, & El‐Elimat, 2007). A 0.2 ml sample was mixed with 0.2 ml Folin–Ciocalteu's reagent at room temperature for 3 min. Then, 0.4 ml of 10% Na_2_CO_3_ was added to the mixture. After standing for 60 min, the absorbance was measured with a spectrophotometer at 725 nm.

The content of total flavonoids was measured according to the NaNO_2_–AlCl_3_–NaOH method (Fu, Xu, Zhao, & Ma, 2006). Briefly, 30 μl of diluted sample (1:2) in methanol was mixed with 9 μl of NaNO_2_ (5%). After reaction for 6 min, 18 μl of AlCl_3_ (10%) was added. Further reaction for 5 min, 60 μl of NaOH (1 M) was added to the mixture. The volume of mixture was adjusted to 300 μl with distilled water and then measured at 510 nm. Different concentrations of rutin (0.1–500 mg/L) were used to calculate the standard curve.

## 3 RESULTS AND DISCUSSION

### 3.1 Sugar content analysis

**Figure 1:**
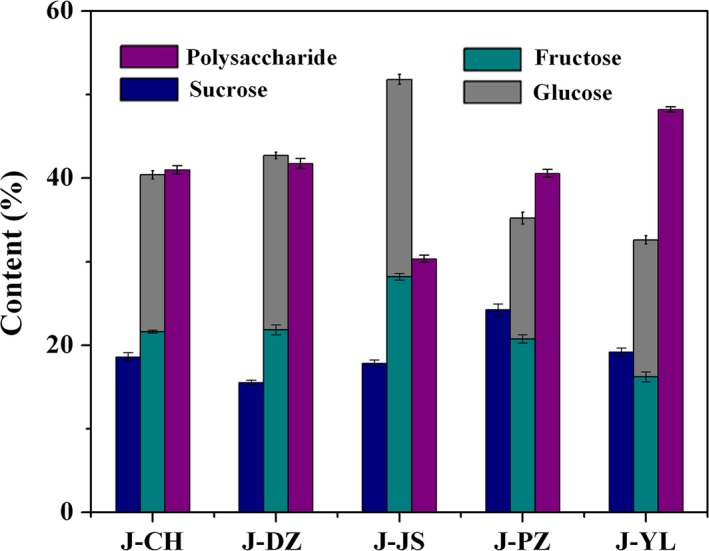
Sugar contents of extracted jujube juice of five different cultivars

It was known that jujube contains high concentration of sugar; however, the content of sugar has obvious difference among varieties (Li et al., 2007). Here, we compared, with content of total sugar, sucrose, reducing sugar in jujube juice of five different cultivars (J‐CH, J‐DZ, J‐JS, J‐PZ, J‐YL), which are widely distributed in China. The content of sucrose in five cultivars was <25%. The content of reducing sugar in cultivar J‐JS reached to 50% which was the highest value among five jujube cultivars (Figure 1). Specially, the content of glucose and fructose in J‐JS reached to 23% and 28%, respectively. The content of sucrose was lower than that of fructose and glucose in J‐JS. The relatively high content of fructose and glucose in J‐JS jujube juice made it a favorable substrate basis for enzymatic synthesis of D‐allulose in later stage.

### 3.2 Bioconversion of D‐allulose in concentrated jujube juice

J‐JS jujube juice contained high content of glucose and fructose which are both high‐calorie sugar. Directly inputting the J‐JS jujube juice in food beverage would increase the risk of obesity, diabetes, and cardiovascular disease (Bocarsly, Powell, Avena, & Hoebel, 2010; Goran, Ulijaszek, & Ventura, 2013; Stanhope et al., 2015). Enzymatic transformation strategy by employment of glucose isomerase (GI), which catalyzed the isomerization of glucose to fructose, and D‐allulose 3‐epimerase (DAE), which catalyzed the epimerization of fructose to D‐allulose, has succeeded in conversion of glucose and fructose in high‐fructose corn syrup (HFCS) to D‐allulose and increased its nutritive value (Men et al., 2014). Here, to obtain one kind of jujube juice containing “healthy sugar,” we attempted to combine GI and DAE to convert glucose and fructose in concentrated jujube juice to D‐allulose. The concentration of D‐glucose and D‐fructose in concentrated jujube was 350 and 360 g/L, respectively (Table 1).

**Table 1:** Concentrations of nutrient substance in jujube juice at different stage

Test item	Initial state	Enzymatic conversion	Fermentation
Glucose (g/L)	352.16 ± 10.23	305 ± 8.26	295 ± 6.32
Fructose (g/L)	360.12 ± 5.32	295 ± 3.19	293 ± 5.69
Allulose (g/L)	n.f.	110 ± 2.10	110 ± 3.09
Lactic acid (g/L)	n.f.	n.f.	3.2 ± 0.20
Viable cells (c.f.u/ml)	n.f.	n.f.	10^8.47^
pH	4.13	6.23	4.02
GABA (mg/L)	0	0	100 ± 0.98
cAMP (mg/L)	114 ± 2.67	116 ± 1.87	116 ± 1.58
Total phenolics (mg/L)	22.25 ± 0.21	21.01 ± 0.13	16.13 ± 0.32
Total flavonoids (mg/L)	146.67 ± 0.34	135.56 ± 0.15	120.12 ± 0.49

Note

n.f.: not found.

**Figure 2:**
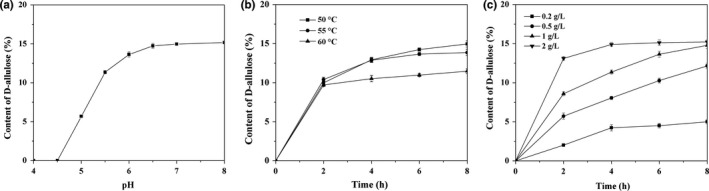
Effect of (a) pH, temperature (b), and enzyme amount (c) on production of D‐allulose

Most often, commercial production of rare sugars is best carried out in acidic conditions. The pH of jujube juice was acidic of 3.6–3.8. Under this condition, both GI and DAE were inactivated (Men et al., 2014; Zhu et al., 2012). Then, we adjusted the pH with 1 M NaOH in a scope ranging from 4.0 to 8.0. The enzymatic conversion under those conditions was measured. The results in Figure 2a showed that the D‐allulose production increased along with improvement of pH value (Figure 2a). When pH was adjusted to 6.5–8.0, the content of D‐allulose among total monosaccharide (glucose, fructose, and D‐allulose) in jujube juice reached to 15.2% which represented the theoretical value (Men et al., 2014).

The effect of temperature on production of D‐allulose was also optimized by control the reaction temperature from 50 to 60°C. When it was controlled at 50°C, the content of D‐allulose reached to 15% after reaction for 8 hr; however, this value decreases to 11.5% under 60°C (Figure 2b). It was probably due to the decrease of enzyme activity of DAE at high temperature (Zhu et al., 2012). It is generally known that an elevated operating temperature is required for the industrial production of rare sugars, because high reaction temperatures induce higher reactivity, lower viscosity, higher stability, higher process yield, and less contamination (Mozhaev, 1993). Nevertheless, operation at temperatures over 70°C accelerates the non‐enzymatic browning reaction and formation of by‐products, and also destroys the nutritional ingredient of fruit juice.

The supplementation of enzyme amount in this reaction was further optimized. We discovered that the synthesis of D‐allulose reached to reaction equilibrium (content of 15.2%) at 8 hr when both enzyme amount of DAE and GI were assigned with 1.0 g/L. Doubling the enzyme amount with 2.0 g/L decreased the reaction time to 4 hr. When enzyme amount decreased to 0.5 g/L or less, more reaction time was needed (Figure 2c).

**Figure 3:**
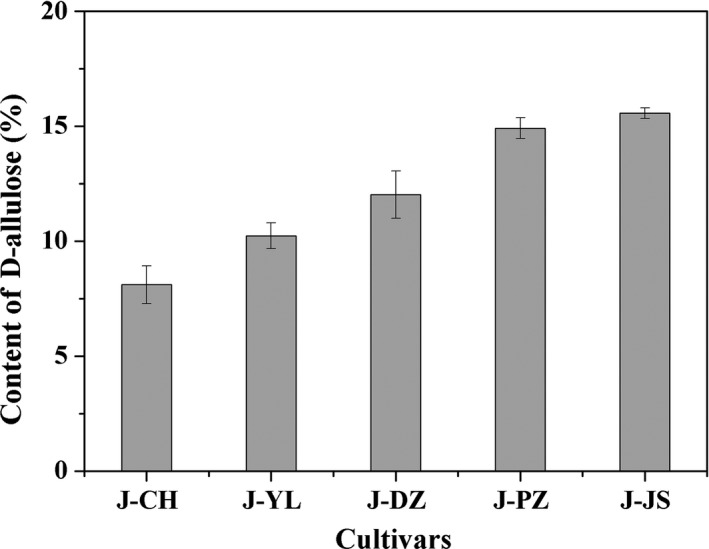
The production of D‐allulose using the jujube juice of five different cultivars

Under the optimal reaction conditions, the concentration of D‐allulose reached to 110 g/L in concentrated jujube juice. Correspondingly, the content of glucose and fructose decreased 13.4% and 18.1%, respectively (Table 1). The jujube juice of other four cultivars was also be transformed. However, the conversion of D‐fructose into D‐allulose varied among all tested cultivars. The component in jujube juice such as trace elements and nutrient substance may inhibit enzyme activity (Li et al., 2007). Specially, the D‐allulose content was the highest in the J‐JS jujube juice, and the content of D‐allulose among total monosaccharide in jujube juice reached to 15% (Figure 3). This value was nearly twofold higher than that in J‐CH (Figure 3). It was reported that the absorption rate of D‐allulose is lower than that of other sweeteners, especially D‐glucose. In addition, D‐allulose could reduce the absorption of dietary D‐fructose and D‐glucose by competitive inhibition of the influx and efflux transporter (Hossain et al., 2015). Moreover, D‐allulose is a reducing sugar, and it could improve food quality through the Maillard reactions. Among all of the D‐ketoses, D‐allulose offered the best overall improvement in the food properties of egg white protein through the Maillard reactions, such as excellent gel strength, emulsifying stability, foaming properties, and antioxidant activities (O'Charoen, Hayakawa, & Ogawa, 2015; Sun, Hayakawa, Ogawa, Fukada, & Izumori, 2008; Zeng, Zhang, Guan, Zhang, & Sun, 2013). As we know, industrial production of D‐allulose has been achieved in Japan and Korea. Recently, a rare sugar syrup containing D‐allulose has been used as a functional food, and can be commercially obtained from Matsutani Chemical Industry Co. Ltd. (Hyogo, Japan) (Zhang, Yu, Zhang, Jiang, & Mu, 2016). In this study, the content of D‐allulose among reducing sugar of jujube juice reached to 15% which indicated that this “healthy sugar” jujube juice showed high application potential in fruit juice beverage market.

### 3.3 Lactic acid bacteria fermentation of Jujube juice containing D‐allulose

**Figure 4:**
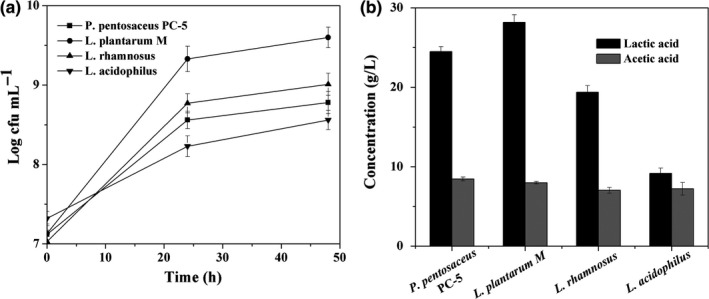
The cell growth (a) and organic acid production (b) of four starters

Microbial fermentation of jujube juice has been applied to develop jujube wine by using *Saccharomyces cerevisiae* and jujube vinegar with *Acetobacter aceti* (Kang, Woo, Lee, & Jeong, 2006; Vithlani & Patel, 2010). Lactic acid bacteria have a long and safe history of application and consumption in the production of fermented foods and beverages. They produce organic acids, mainly lactic acid, which cause rapid acidification of the raw material and enhance shelf life and microbial safety. Also, their production of acetic acid, aroma compounds, GABA, free amino acids, and exopolysaccharides is of importance in organoleptic, nutritional, or health advantages. In this study, lactic acid bacteria fermentation of jujube juice containing D‐allulose was carried out to metabolize glucose and accumulate nutrition component, increasing the nutritional value of jujube juice. Four starters including *P. pentosaceus* PC‐5, *L. plantarum* M, *L. rhamnosus*, and *L. acidophilus* were selected. Initially, the cell growth and organic acid production of those strains were compared on MRS medium. After fermentation for 48 hr, the viable cells of *L. plantarum* M reached to 10^9.6^ c.f.u/ml from 10^7.1^ c.f.u/ml, which was the highest value among four starters (Figure 4a). The lactic acid production of *L. plantarum* M and *P. pentosaceus* PC‐5 was 28.2 and 24.5 g/L, respectively (Figure 4b). Those results showed those two starters are suitable candidate for jujube juice fermentation. Then, the mixed cultures of *L. plantarum* M and *P. pentosaceus* PC‐5 were investigated to ferment the enzymatic transformed jujube juice. After fermentation for 24 hr, the cell densities reached to 10^8.5^ c.f.u./ml from 10^7.2^ c.f.u/ml, and the pH decreased to 4.02 from 6.23. This fermentation consumed 10 g/L glucose and produced 3.2 g/L lactic acid (Table 1). Those results showed that lactic acid bacteria have the ability to grow under the stressful condition of high concentrated sugars in jujube juice.

### 3.4 Analysis of the nutritional component after fermentation

It is noteworthy that minerals are important for human nutrition (Ekmekcioglu, 2000). We then measured the mineral substance in jujube juice before and after fermentation. The results showed that potassium (K^+^) content was 6,388.64 ± 62.31 mg/kg, which presented the predominant mineral in jujube juice. The content of magnesium (Mg^2+^) was 442.86 mg/kg. It was required for many enzymes, especially for the kinase families that catalyze ATP‐dependent phosphorylation reactions (Li et al., 2007). Calcium (Ca^2+^), which shows biological activity in lowering blood pressure (Zemel, 1997), was also detected in jujube juice. After fermentation for 24 hr, content of iron (Fe^2+^) and zinc (Zn^2+^) increased by 34% and 20.7% compared with the initial state, respectively. The metal ion Zn^2+^ present in high concentrations in ocular tissue, particularly in retina and choroid, showed many biological activities for eyes which were believed to interact with taurine and vitamin A, modify photoreceptor plasma membranes, regulate the light–rhodopsin reaction, modulate synaptic transmission, and serve as an antioxidant. (Bruce, Grahn, Phyllis, Katherine, & Zhen, 2015). The increase of Fe^2+^ content would improve the blood tonic function of jujube juice (Marcel et al., 1999). Other minerals were almost unchanged (Table 2).

**Table 2:** The difference of trace elements between the initial and fermentation state

Test item	Unit	Initial state	Fermentation
Fe	mg/kg	2.93 ± 0.11	3.93 ± 0.23
Mg	mg/kg	442.86 ± 7.17	448.90 ± 6.34
Mn	mg/kg	1.49 ± 0.11	1.77 ± 0.09
Ca	mg/kg	82.20 ± 0.57	86.74 ± 0.69
Zn	mg/kg	5.3 ± 0.31	6.4 ± 0.42
K	mg/kg	6,388.64 ± 62.31	6,414.68 ± 83.26
P	mg/100 g	60 ± 1.25	69.9 ± 0.97

**Figure 5:**
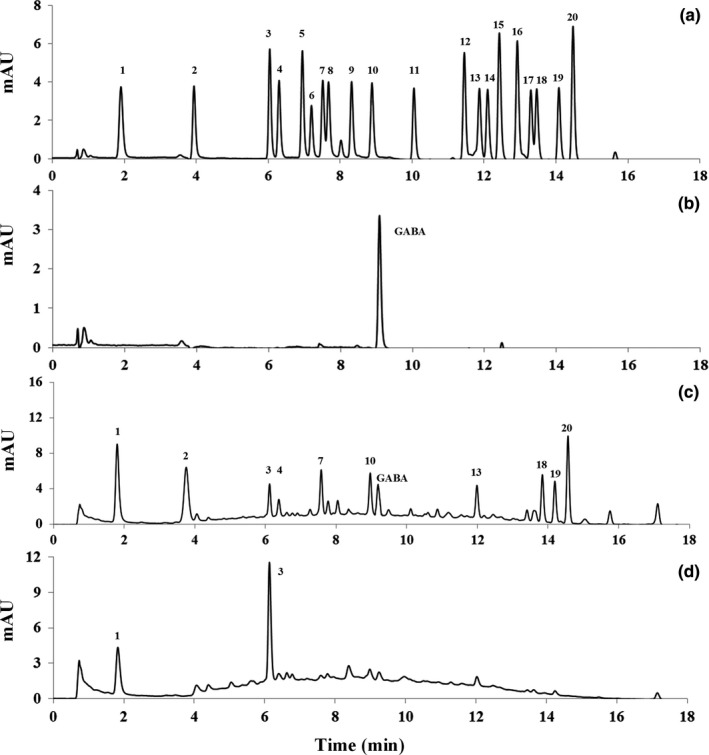
HPLC analysis of free amino acids and GABA. (a) The standard of 20 amino acid including: 1. Asp; 2. Glu; 3. Asn; 4. Ser; 5. Gln; 6. His; 7. Gly; 8. Thr; 9. Arg; 10. Ala; 11. Tyr; 12. Cy2; 13. Val; 14. Met; 15. Nva; 16. Trp; 17. Phe; 18. Ile; 19. Leu; and 20. Lys; (b) The standard of GABA; (c) Sample after enzymatic transformation and fermentation; and (d) Sample in initial state

Next, we investigated the effect of fermentation on change of free amino acids (FAAs) component in jujube juice. After fermentation for 24 hr, FAAs component was obviously different from the initial state. Typically, the content of Asp, Glu, Ser, Gly, Thr, Ala, Val, Ile, and Leu increased at different level (Figure 5). Branched‐chain amino acids (BCAAs), such as Val, Ile, and Leu, are necessary for human health. Supplementation of BCAA‐rich diets showed positive effects on regulation of body weight, muscle protein synthesis, and glucose homeostasis, and they are important and effective nutritional supplement for sport. (Lynch & Adams, 2014). Specially, Asp has a protective effect on myocardium. It reduces the amount of nitrogen and carbon dioxide in the blood and enhances the liver function and eliminates fatigue (Thomas et al., 2003). The production of FAAs was probably derived from the decomposition of proteins or small peptides which was synthesized from the lactic acid bacteria fermentation of glucose and was more easily absorbed by the body in drinks.

Lactic acid bacteria like *L. plantarum* contain glutamate decarboxylase which catalyzes the decarboxylation of Glu to GABA (Siragusa et al., 2007). GABA is an inhibitory neurotransmitter and has noticeable tranquilizing bioactivity (Wong et al., 2003). The development of GABA‐enriched foods and beverages is in high demand. In this study, the content of Glu dramatically increased compared with the initial state in the fermented jujube juice (Figure 5). It showed that GABA could be detected after fermentation using mixed cultures of *L. plantarum* and *P. pentosaceus* PC‐5. As expected, we detected 100 mg/L GABA in the jujube juice after fermentation for 24 hr (Figure 5). GABA has been reported to lower blood pressure in experimental animals and human subjects. Previous studies showed that the ingestion of GABA decreased systolic blood pressure in spontaneously hypertensive rats and the hypotensive activity of GABA was dose‐dependent from 0.05 to 5.00 mg/kg (Hayakaw et al., 2004; Kimura, Hayakawa, & Sansawa, 2002). A previous study showed that the oral administration of GABA in rice germ of 26.4 mg daily was effective in treating neurological disorders (Okada et al., 2000). The GABA enrichment in jujube would upgrade its value and show high application potential in food and beverage industry.

**Figure 6:**
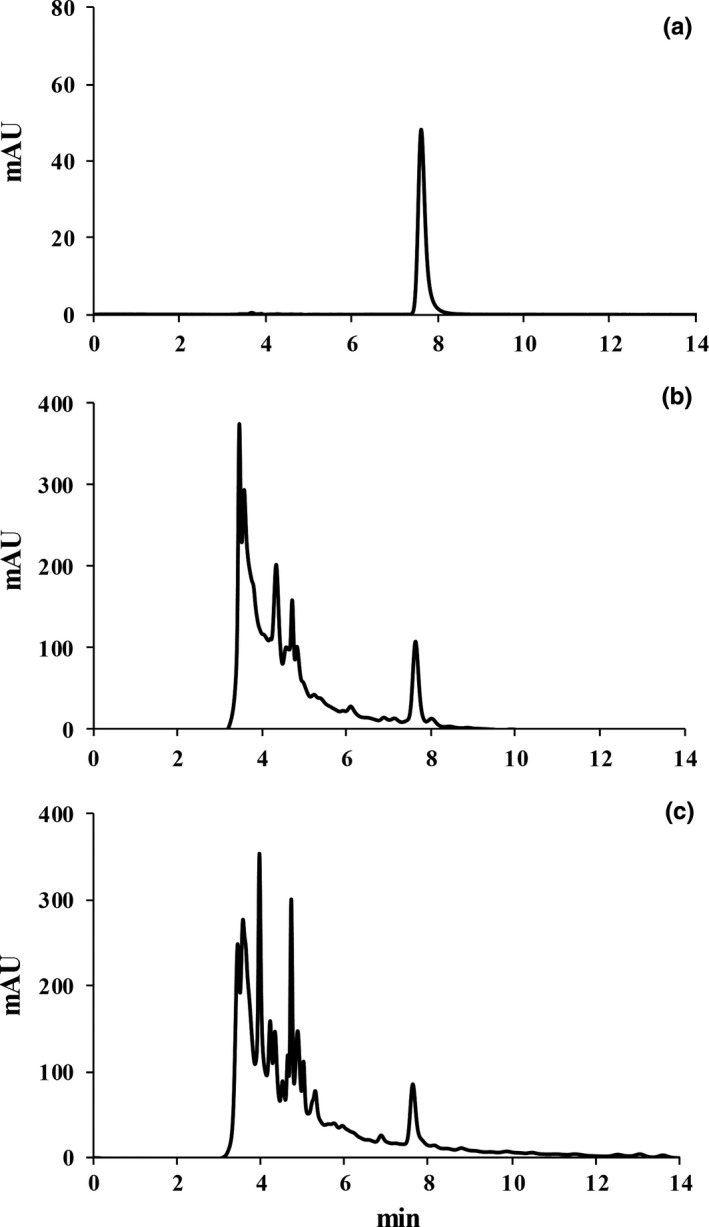
HPLC analysis of cyclic adenosine monophosphate (cAMP). (a) The standard of cAMP; (b) Sample of initial state; and (c) Sample after enzymatic transformation and fermentation

The cAMP is an important nutrient substance in jujube juice (Kou et al., 2015). We detected 114 mg/L cAMP in extracted jujube juice (Figure 6). Its content did not change after enzyme transformation and lactic acid bacteria fermentation (Table 1). The content of total phenolics and flavonoids generally influences the biological activity such as antioxidant and anti‐inflammatory activities (Matias et al., 2014; Timmers et al., 2015). We then compared the content of total phenolics and flavonoids during enzymatic transformation and lactic acid bacteria fermentation process. The total phenolics and total flavonoids in jujube juice were about 22.76 mg/L and 146.67 μg/L, respectively. Enzymatic transformation did not alter the level of total phenolics and flavonoids compounds; however, fermentation decreased the total phenolics and flavonoids to 16.1 mg/L and 120.1 μg/L, respectively (Table 1). It was probably due to the metabolic capacity of lactic acid bacteria used in degradation of some phenolic or flavonoid compounds such as oleuropein (Landete, Curiel, Rodríguez, de las Rivas, & Muñoz, 2008) and food phenolic acids (Rodríguez, Landete, de las Rivas, & Munõz, 2008). Further study should be made to select other lactic acid bacteria which showed low or none metabolic capacity to phenolic or flavonoid compounds.

## 4 CONCLUSION

With the improvement of people's living standard and health awareness, green and healthy food is generally welcomed by consumers. Compared with other healthy food, juice is easy to be accepted in price, mainly as a drink to meet the needs of consumers. However, fruit juices are high in high‐energy sugars such as glucose and fructose. In this study, we coupled enzymatic transformation and lactic acid bacteria fermentation to increase the nutritional value of jujube juice. The concentration of non‐calorie D‐allulose reached to 110 g/L, accounts for 15% of all reducing sugar, which expanded the number of people who drink juice to promote greater health. The mixed cultures of two lactic acid bacteria were introduced into enzymatic transformed jujube juice. This fermentation accumulated 100 mg/L GABA, which has neurotransmission, hypotension, diuretic, and tranquilizer effects, and increased the content of BCAAs, which could promote insulin and growth hormone release and many free amino acids (Asp, Glu, Gly, and Ala) at different level, but did not change the concentration of native functional components such as cAMP. Moreover, this fermentation increased the content of iron (Fe^2+^) and zinc (Zn^2+^). The value‐added jujube juice might serve as a low‐calorie and probiotic functional beverage and show high application potential in food industry. In addition, this producing process paves the way to develop other functional beverage based on the fruit juice which contained high content of glucose and fructose.

## CONFLICT OF INTEREST

The authors declare that they do not have any conflict of interest.

## ETHICAL REVIEW

This study does not involve any human or animal testing.
